# Supplementary material for: How Carbon Nanoparticles, Arbuscular Mycorrhiza, and Compost Mitigate Drought Stress in Maize Plant: A Growth and Biochemical Study
Source: Plants (Basel). 2022 Dec 1;11(23):3324. doi: 10.3390/plants11233324 (PMC9739014; doi:10.3390/plants11233324)
Supplement: Supplementary file 1 [file plants-11-03324-s001.zip › plants-2006350-supplementary.pdf]

Table S1: Variations in rainfall and relative humidity in the research area on a monthly basis. The information was gathered from the Jeddah meteorological station in Saudi Arabia. Mx stands for maximum temperature, Mn for minimum temperature, and M stands for mean temperature during study period (January to December 2021).

	Temperature (C°)			Relative Humidity (%)	Precipitation (mm)
	Mx	Mn	M		
January	26.8	18.2	22.3	81	17
February	28	19.6	23.6	87	10
March	30.5	20.7	25.3	95	27
April	32.8	23.2	27.8	83	10
May	35.9	26.8	31.2	84	30
June	37.9	27.3	32.3	86	3
July	38.5	29.1	33.6	93	31
Augustus	38.7	30	34.2	81	1
September	38.7	30	33.9	88	11
October	39.2	27	32.4	89	1
November	33.1	25.3	28.8	88	20
December	31.9	22.4	26.6	84	8

Table S2. Physicochemical properties of soil used in 2021 growing seasons. ± Standard deviation (4 replicates).

Property	Control	Composite	Mycorrhiza	Com/Myco
**Particle size distribution (%)**				
Sand	77±3.5	75±3.1	74±3.5	74±4.3
Silt	6±0.5	7.8±0.5	7.2±0.7	8±0.5
Clay	16±0.8	17±0.8	18.5±0.8	18±0.8
**Physical and chemical analysis**				
pH	7.61±0.87	7.72±0.4	7.55±0.54	7.25±0.34
OM %	1.25±0.13	3.85±0.13	2.15±0.33	4.45±0.38
EC (ds m^{-1})	0.57±0.74	1.78±0.7	1.68±0.1	2.28±0.14
**Cations (µg/gm)**				
Ca^{++}	1.14±0.16	7.5±0.83	4.54±0.53	9.54±0.63
Mg^{++}	0.80±0.2	4.54±0.17	2.70±0.38	5.6±0.41
Na^{+}	1.43±0.03	8.3±0.9	7.6±0.8	7.41±0.6
K^{+}	3.29±0.13	9.34±0.5	4.25±0.5	11.81±0.8
**Anions (meq L^{-1})**				
CO_3^{-}	1.10±0.2	11.1±0.6	3.10±0.1	14.5±0.9
HCO_3^{-}	2.18±0.16	7.18±0.6	3.18±0.4	8.18±0.1
Cl^{-}	2.6±0.1	12.2±0.1	3.2±0.6	12.6±0.2
SO_4^{-}	1.55±0.3	6.7±0.3	2.05±0.6	6.51±0.2
**Available nutrients (µg/gm)**				
N	26.5±6	116.1±7	76.59±8	186.5±12
P	1.81±0.8	16.81±4	6.81±0.8	14.81±1.2
K	79±27	299±24	149.8±23	369±21
**Micro-nutrients (µg/gm)**				
Fe	1.1±0.7	2.2±0.7	3.21±4	8.94±1.2
Mn	2.1±0.8	7.5±1.1	5.54±1.1	11.4±0.9
Zn	0.21±0.0	0.55±0.1	0.95±0.04	1.55±0.1
Cu	0.4±0.01	1.94± 0.1	1.14± 0.1	3.94± 0.2
